# Reliable Gas Phase Reaction Rates at Affordable Cost by Means of the Parameter-Free JunChS-F12 Model Chemistry

**DOI:** 10.1021/acs.jctc.3c00343

**Published:** 2023-05-31

**Authors:** Vincenzo Barone, Jacopo Lupi, Zoi Salta, Nicola Tasinato

**Affiliations:** SMART Laboratory, Scuola Normale Superiore di Pisa, Piazza dei Cavalieri 7, 56125 Pisa, Italy

## Abstract

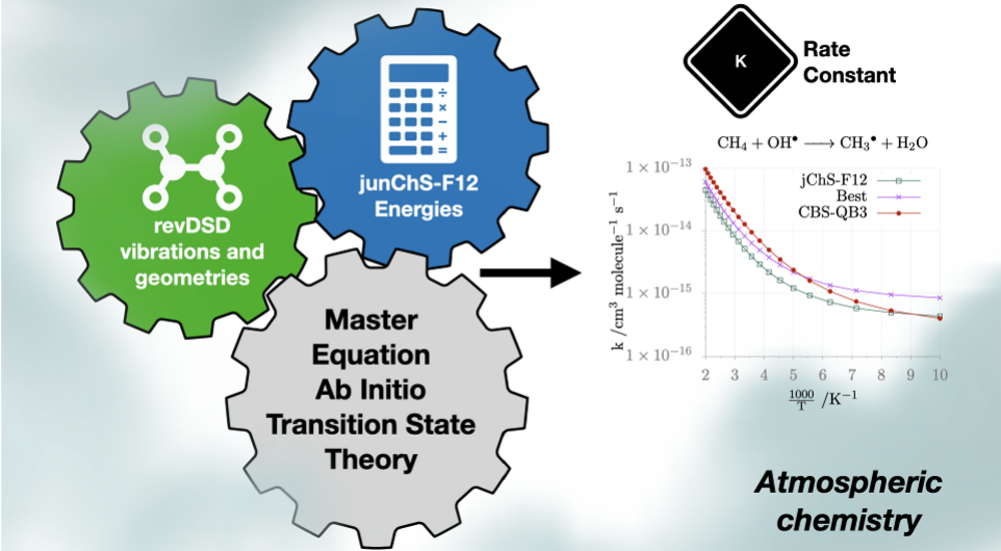

A recently developed strategy for the computation at affordable cost of reliable barrier heights ruling reactions in the gas phase (junChS, [Barone, V.; J. Chem. Theory Comput.2021, 17, 4913−49283422893510.1021/acs.jctc.1c00406PMC8359010]) has been extended to the employment of explicitly correlated (F12) methods. A thorough benchmark based on a wide range of prototypical reactions shows that the new model (referred to as junChS-F12), which employs cost-effective revDSD-PBEP86-D3(BJ) reference geometries, has an improved performance with respect to its conventional counterpart and outperforms the most well-known model chemistries without the need of any empirical parameter and at an affordable computational cost. Several benchmarks show that revDSD-PBEP86-D3(BJ) structures and force fields provide zero point energies and thermal contributions, which can be confidently used, together with junChS-F12 electronic energies, for obtaining accurate reaction rates in the framework of the master equation approach based on the ab initio transition-state theory.

## Introduction

The main focus of atmospheric chemistry is the descritption and analysis of Earth’s atmosphere in terms of the underlying physical-chemical processes controlling the sources and fate of the different chemical species produced by natural or anthropogenic emissions. However, despite significant progress, the interpretation of atmospheric processes in terms of the underlying chemistry faces against a number of difficulties mainly related to the interplay between chemical composition and meteorological/transport processes.

In the last years, the increasing synergism among the major pillars of atmospheric chemistry, namely observational measurements, laboratory investigation, and atmospheric modeling,^1,2^ is providing invaluable insights into the intricate phenomena occurring in the atmosphere. In this framework, computational chemistry can be of considerable help for gaining additional information about ground and excited state properties of chemical species, their photochemical pathways, chemical reaction mechanisms, and rate coefficients.^3^ In particular, kinetic and mechanistic features of reactions are usually interpreted employing the Arrhenius equation to describe the variation of the rate constant with temperature^4^
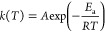
1where *A* is the pre-exponential or frequency factor (which may involve a small dependence on temperature) and *E*_a_ is the activation energy. While more precise definitions are available,^5,6^ the activation energy is usually interpreted as the minimum energy (kinetic plus potential, relative to the lowest state of reactants) that reactants must have to form products and the pre-exponential factor is a measure of the rate at which collisions occur. If a reaction obeys the Arrhenius equation, then a plot of ln *k* versus  should produce a straight line, whose slope and intercept at the origin are  and *A*, respectively. However, many reactions of wide current interest do not obey the Arrhenius equation and/or have negative activation energies (rate constants that decrease when the temperature is increased).^7,8^ At the same time, the experimental characterization of several reactions of atmospheric interest is made difficult by the involvement of highly reactive species, such as free radicals or ions.

Under such circumstances, accurate yet feasible quantum chemical approaches are needed. The main factors determining the accuracy of computed rate constants are the reaction energies and the energy barriers for all the elementary steps involved in the reaction under investigation. In the absence of species with a strong multireference character and/or nonadiabatic effects, the coupled cluster (CC) approach delivers accurate results provided that the most important classes of excitations are included together with complete basis set (CBS) extrapolation, core–valence (CV) correlation, and, if needed, other minor effects (scalar relativistic, diagonal non-Born–Oppenheimer, spin–orbit). Due to an effective error compensation, single, double, and (perturbative estimates of) triple excitations are usually sufficient, leading to the CCSD(T)-CBS+CV model, which is often considered the gold standard of contemporary computational chemistry. At this level, chemical accuracy (4 kJ mol^–1^) can be reached either by employing large basis sets,^9^ resorting to empirical parameters in conjunction with smaller basis sets (e.g., G4,^10^ CBS-QB3^11^), or employing explicitly correlated (F12) models (e.g., W1-F12^12^ or SVECV-f12^13^). The most reliable protocols (e.g., HEAT,^14^ W4,^12^ and their explicitly correlated HEAT-F12 and W4-F12^15^ variants) further increase the overall accuracy (below 1 kJ mol^–1^) including additional (expensive) contributions. In this connection it should be pointed out that the latter protocols push also geometry optimizations to the limit, whereas, at the other extreme, G4 and CBS-QB3 schemes employ B3LYP geometries, whose accuracies are often unsatisfactory.^10,16^

Next, zero point energies (ZPEs) and finite temperature contributions (FTCs) come into play, which are determined by geometries and vibrational frequencies. In this connection, effective approaches going beyond the standard rigid rotor/harmonic oscillator (RRHO) model are needed especially when light atoms or hindered rotors are involved. Finally, barrierless entrance or exit channels are often encountered for reactions in the gas phase, which require the accurate description of noncovalent interactions.

Based on these premises, we have developed a composite method, referred to as the “cheap” scheme (ChS) and devoid of any empirical parameter, which has provided accurate structural and energetic data at nonprohibitive costs.^17−19^ In conjunction with geometries and harmonic frequencies computed by double-hybrid functionals, ChS has given promising results also for the activation energies of some reactions of astrochemical interest.^20−24^ In analogy with the W1X^25^ and SVECV-f12^13^ composite methods, ChS employs the second order Møller–Plesset perturbation theory (MP2)^26^ for estimating the CV correlation. A further reduction of the computational cost is achieved by performing, in accord with the correlation consistent composite approach (ccCA),^27,28^ also the CBS extrapolation at the MP2 level. Quite recently, an improved variant (referred to as the jun-Cheap scheme, junChS) has been introduced, which, thanks to the use of the “june” partially augmented basis sets of the “calendar” family,^29^ provides accurate results also for noncovalent interactions^30,31^ and activation energies.^16^

In the present paper we perform a comprehensive benchmark of the latest member of the “cheap” family of composite methods, junChS-F12,^32,33^ for several classes of reactions for which accurate reference results are available or have been purposely computed. We will show that, thanks to the replacement of conventional post-Hartree–Fock methods by their explicitly correlated (F12) counterparts, this model chemistry improves the accuracy of previous variants, strongly reducing the uncertainty of CBS extrapolation without any excessive increase of computational requirements. Together with electronic energies, we analyze also the roles of geometries, ZPEs, and FTCs in tuning reaction rates in the framework of the master equation approach based on the ab initio transition-state theory (ME/AITST).^34−36^

## Computational Details

All the composite schemes discussed in the present work employ the cc-pV(n+d)Z (hereafter nZ)^37^ or jun-cc-pV(n+d)Z (hereafter jnZ)^29^ families of basis sets.

The geometrical parameters and harmonic vibrational frequencies of energy minima and first-order saddle points (transition states) are obtained employing analytical gradients and Hessians computed by the revDSD-PBEP86-D3(BJ) double-hybrid functional^38,39^ in conjunction with the j3Z basis set (this combination of functional and basis set will be referred to in the following as rDSD).

At those geometries, single point energy evaluations are performed by the explicitly correlated coupled cluster method, including single, double, and (perturbatively) triple excitations (CCSD(T)-F12)^40,41^ within the frozen-core approximation and in conjunction with the j3Z basis set. Next, CBS extrapolation, CV correlation, and, possibly, other minor terms are added at different levels depending on the specific model chemistry. Finally, the experimental values of spin–orbit couplings are employed for O, OH, SH, and Cl radicals, lowering their electronic energies by 0.9, 0.8, 2.3, and 3.5 kJ mol^–1^, respectively.^42^

All the DFT computations have been performed with the Gaussian code,^43^ F12 calculations with the Molpro package^44^ and CCSDT or CCSDT(Q) energy evaluations with the MRCC program.^45^ Finally, diagonal Born–Oppenheimer Corrections (DBOC) and relativistic contributions have been computed by the CFOUR code.^46^

### The junChS-F12 Model Chemistry

The junChS-F12 total electronic energies are obtained by the following recipe:
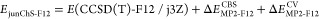
2where
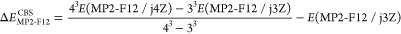
3and
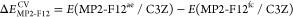
4In the above equations Δ*E*_MP2-F12_^CBS^ is the MP2-F12 correlation energy extrapolated to the CBS limit using the n^–3^ formula^47^ and Δ*E*_MP2-F12_^CV^ is the MP2-F12 energy difference between all electron (ae) and frozen core (fc) calculations employing the cc-pwCVTZ basis set (C3Z).^48^ At this level, the extrapolation of Hartree–Fock (HF) and correlation contributions is performed with the same equation and basis sets since several tests have shown that this simplified recipe has a negligible impact on the overall accuracy of the results.^27,28,30,32^

Derivation of eq 2 w.r.t. Cartesian coordinates leads to the junChS-F12 version of the so-called “gradient scheme” introduced by Gauss and co-workers^49,50^ for geometry optimizations by composite methods
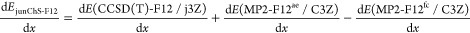
5where, on the grounds of previous experience,^32,51^ the CBS contribution can be safely neglected. To further extend the applicability of composite approaches to larger molecules, an effective solution is provided by the so-called “geometry” scheme.^52,53^ This is based on the assumption that the additivity approximation can be directly applied to geometrical parameters and only requires geometry optimizations at several levels of theory. The different contributions are thus evaluated separately and then combined together. This approach will be used in the following sections to analyze the role of different geometries on the final evaluation of electronic energies (ΔGEOM contribution).

### Additional Terms

Starting from junChS-F12 electronic energies, additional terms can be added to improve the accuracy of the final results (*E*_Best_):
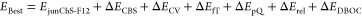
6The CBS and CV contributions refer to the differences between evaluations of these terms at the CCSD(T)-F12 and MP2-F12 levels. The diagonal Born–Oppenheimer correction Δ*E*_DBOC_^54−57^ and the scalar relativistic contribution to the energy Δ*E*_rel_^58,59^ are computed at the HF-SCF/aug-cc-pVDZ and CCSD(T)/aug-cc-pCVDZ levels, after having checked their convergence with respect to contributions calculated with triple-ζ basis sets for a few stationary points. Finally, the corrections due to full treatment of triple (Δ*E*_fT_) and perturbative treatment of quadruple (Δ*E*_pQ_) excitations are computed, within the fc approximation, as energy differences between CCSDT and CCSD(T) and between CCSDT(Q) and CCSDT calculations employing the cc-pVTZ and cc-pVDZ basis set, respectively.

In the following, the method obtained including only the first three terms of eq 6 will be referred to as CBS+CV, whereas the method including all the terms of eq 6 will be referred to as *Best*. While straightforward generalizations of eq 4 would allow geometry optimizations at the CBS+CV and *Best* levels, this route has not been pursued here due to the negligible improvement over junChS-F12 in the former case and the lack of analytical gradient implementations for fT and pQ contributions in the latter case.

### Zero Point Energy and Finite Temperature Contributions

Accurate determination of thermochemical and kinetic parameters by quantum chemical methods requires, in addition to electronic energies, also zero point energies (ZPE) and finite temperature contributions (FTC), which are usually obtained within the RRHO approximation, possibly employing empirical scaling factors.^60^ In the present context, the use of empirical factors is avoided by resorting to generalized second order vibrational perturbation theory in conjunction with a separate treatment of large amplitude motions.^61,62^ In fact, a resonance-free expression for ZPEs of energy minima and transition states,^63,64^ an unsupervised smoothing procedure (HDCPT2) for fundamental frequencies^65^ and a fully unsupervised detection and treatment of torsional motions (hindered rotor, HR, approximation)^66^ have been implemented in the Gaussian code^43^ and validated.^67^ As a consequence a fully black-box procedure is available for taking into account all these contributions.

Next, partition functions can be computed by the so-called simple perturbation theory (SPT),^68^ which retains the formal expression of the harmonic partition function, but employing the anharmonic ZPE and fundamental levels (Δ_*i*_) issuing from HDCPT2 and HR computations.
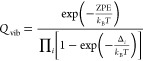
7This approximation provides results in remarkable agreement with accurate reference values and leads to analytical expressions for the different thermodynamic functions.^68^

### Kinetic Models

Global and channel-specific rate constants can be computed solving the multiwell one-dimensional master equation using the chemically significant eigenvalues (CSEs) method within the Rice–Ramsperger–Kassel–Marcus (RRKM) approximation, as implemented in the MESS code.^41^ The collisional energy transfer probability is described using the exponential down model^69^ with a temperature dependent Δ*E*_down_ of 260 × (*T*/298)^0.875^ cm^–1^ in an argon bath gas.^69^

For channels ruled by a distinct saddle point, rate coefficients are determined by conventional transition state theory (TST)^70^ including tunneling as well as non classical reflection effects by the Eckart model.^71^ Instead, rate constants for barrierless elementary reactions are computed by the phase space theory (PST).^72,73^ The isotropic attractive potential *V*_eff_ entering the PST is described by a  power law, whose *C* coefficient is obtained by fitting rDSD energies computed at various long-range distances between the fragments.

While the adopted models for the inclusion of tunneling and the description of barrierless entrance channels usually deliver qualitatively correct results, they neglect a number of effects (e.g., variational location of the TS, nonvanishing curvature of the reaction path, etc.), whose proper treatment would require more advanced models.^21,74^ However, these models require, in turn, additional information besides the characterization of the stationary points governing each elementary step. As a consequence, we prefer to postpone these aspects after the reliability of the proposed approach for the structural and energetic properties of stationary points has been definitely assessed.

The rate constants of the overall reactions evaluated at different temperatures are fitted by the three-parameter modified Arrhenius equation proposed by Kooij:^75,76^
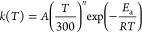
8where *A*, *n*, and *E*_a_ are the fitting parameters, and *R* is the universal gas constant.

## Results and Discussion

The most widely employed reference results for reaction barriers are collected in the DBH24 compilation^77,78^ containing results mostly obtained at the CCSDTQ5/CBS level by means of the W4 composite method^79^ for a statistically representative set including 3 prototypes for each of the following classes of reactions: heavy atom transfer, nucleophilic substitution, unimolecular and association reactions, and hydrogen-transfer reactions.

Table 1 compares the reaction barriers computed at the junChS-F12 level to the reference values of ref (78). From a technical point of view, the results show that F12a and F12b variants of the CCSD-F12 method^41^ provide comparable results, so that only F12a values will be discussed in detail in the following. As shown in Table 1, the CCSD(T)-F12/j3Z error is already on par with the best available composite methods^13^ and is further slightly reduced adding CBS and CV contributions by inexpensive MP2-F12 computations. These trends confirm that two-point extrapolation at the MP2-F12 level is an effective route for estimating the CBS limit without introducing additional computational bottlenecks with respect to the underlying CCSD(T)-F12/j3Z reference. As a matter of fact, already for reactions involving two heavy atoms, junChS-F12 computations require no more than twice the time of the CCSD(T)-F12/jun-cc-pVTZ step and are 1 order of magnitude faster than their CBS+CV counterparts. It is also remarkable that all the energy barriers showing non-negligible errors have quite large contributions from full triple and perturbative quadruple excitations (fT+pQ), which are not included in the junChS-F12 approach nor in its CBS+CV counterpart. Table 1 collects also the differences between anharmonic and harmonic ZPE contributions to energy barriers (Δanh). While these terms (together with spin–orbit contributions) will be discussed in more detail in the following, we already point out that their contribution is sometimes comparable with that of (fT+pQ).

**Table 1:** Theoretical Values of the Barrier Heights (Not Including Spin–Orbit Correction and ZPE) in the DBH24 Compilation Obtained at Different Levels of Theory^a^

		forward/reverse barrier height				
	reactions	CC-F12a/j3Z	junChS-F12	fT+pQ	ref (78)	Δanh
heavy-atom transfer						
a1	H^•^ + N_2_O → OH^•^ + N_2_	74.0/347.8	73.9/348.3	–1.4/–3.2	71.7/345.0	–0.2/–0.2
		[74.2/347.4]				
a2	H^•^ + ClH → HCl + H^•^	75.2/75.2	73.6/73.6	–0.55/–0.5	75.3/75.3	1.3/1.3
		[75.9/75.9]				
a3	CH_3_^•^ + FCl → CH_3_F + Cl^•^	29.9/250.4	29.9/251.3	–1.0/–1.2	28.2/251.0	–0.8/0.1
		[30.0/250.5]				
nucleophilic substitution						
a4	Cl^–^···CH_3_Cl → ClCH_3_···Cl^–^	56.2/56.2	56.2/56.2	–1.0/–1.0	56.1/56.1	0.1/0.1
		[56.7/56.7]				
a5	F^–^···CH_3_Cl → FCH_3_···Cl^–^	14.3/122.5	14.6/123.7	–0.8/–0.9	14.4/123.1	0.0/0.0
		[14.5/123.7]				
a6	OH^–^ + CH_3_F → HOCH_3_ + F^–^	–11.8/73.5	–8.9/74.8	–1.3/–1.4	–10.2/73.9	–0.4/0.0
		[−11.1/74.0]				
unimolecular and association						
a7	H^•^ + N_2_ → HN_2_^•^	61.4/45.8	60.8/46.3	0.5/0.7	60.1/44.4	–0.4/0.1
		[61.8/45.4]				
a8	H^•^ + C_2_H_4_ → C_2_H_5_^•^	9.1/177.1	8.3/176.6	–0.5/–0.6	7.2/174.7	–0.3/–0.2
		[9.3/177.0]				
a9	HCN ↔ HNC	199.6/137.8	201.2/138.6	–0.2/–0.6	201.1/137.3	0.1/0.1
		[199.3/137.5]				
hydrogen transfer						
a10	OH^•^ + CH_4_ → CH_3_^•^ + H_2_O	27.1/82.2	27.7/83.3	–0.7/–0.6	28.1/82.0	1.0/0.9
		[27.1/81.9]				
a11	H^•^ + OH^•^ →H_2_ + ^3^O	46.3/56.9	46.6/57.6	–0.6/–1.0	44.8/54.9	0.7/0.8
		[45.8/56.6]				
a12	H^•^ + H_2_S → H_2_ + HS^•^	17.1/73.9	16.0/76.3	–0.5/–0.4	15.2/72.5	–0.5/–0.6
		[17.6/73.6]				

a F12b values are reported in square brackets. The contributions of full-triple and perturbative-quadruple excitations (fT+pQ) and the differences between anharmonic and harmonic ΔZPEs computed at the rDSD level (Δanh) are also given. All the values are in kJ mol^–1^.

Two larger compilations of energy barriers are available for prototypical reactions involving the transfer of hydrogen (HTBH38/08^80^) and non-hydrogen atoms (NHTBH38/08^81^), respectively. However, the values not already included in the DBH24 set have been obtained at a lower computational level (W1^82^). In order to investigate the role of different effects on energy barriers we have computed *Best* values for all the reactions belonging to those two sets. It is noteworthy that rDSD energy barriers, although not directly used in the junChS model chemistries, show mean unsigned errors (MUEs) smaller than 8.0 kJ mol^–1^, thus suggesting that the corresponding geometries should be sufficiently accurate for single-point energy evaluations at higher computational levels.

**Figure 1:**
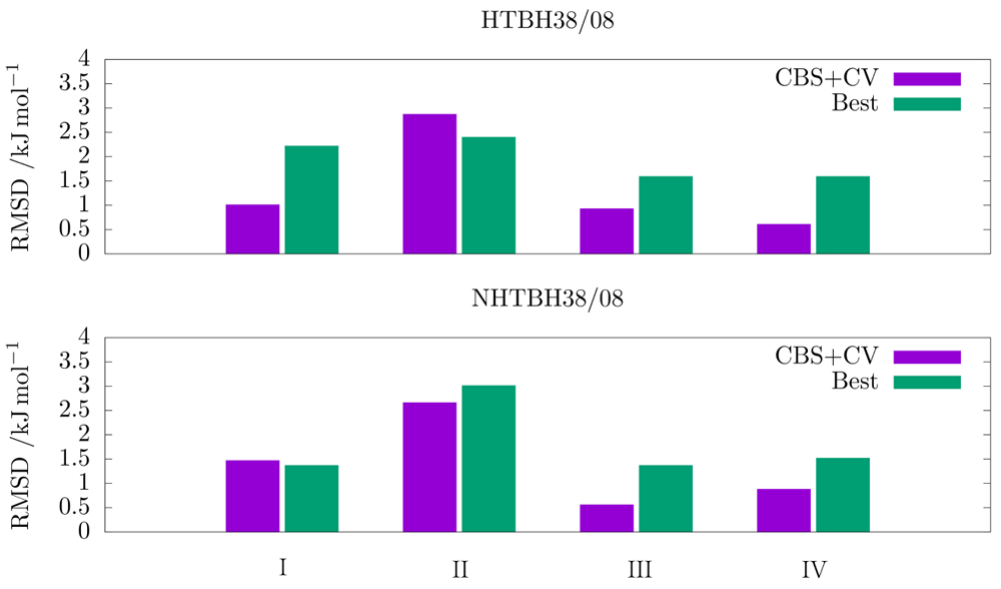
Root-mean-square deviations of different model chemistries from reference values (CBS+CV or *Best*) of energy barriers belonging to the HTBH38/08 and NHTBH38/08 compilations: junChS (I), junChS without MP2 CBS extrapolation (II), junChS-F12 (III) and junChS-F12 without MP2-F12 CBS extrapolation (IV).

Figure 1 shows the errors issued from different model chemistries, whereas the corresponding energy barriers are given in Table S1 of the Supporting Information (SI). It is quite apparent that CBS extrapolation plays a much more important role in conventional composite methods (junChS) than in their explicitly correlated counterparts (junChS-F12). However, also in the latter case its inclusion (together with that of the CV contribution) is surely warranted in view of the quite negligible cost. As expected, the junChS-F12 model shows smaller errors with respect to the reference values than the junChS approach, with both models clearly satisfying the requirements of chemical accuracy (i.e., errors within 4 kJ mol^–1^) without the need of any empirical parameter.

Tables 2 and 3 show the contributions given by improved geometries (junChS-F12 vs rDSD referred to as Δ*G*EOM), core–valence correlation (CV–F12), triple excitations (fT), quadruple excitations (included perturbatively, pQ), diagonal Born–Oppenheimer corrections (DBOC), scalar relativistic contributions (rel), and spin–orbit couplings (Δ*S*O). The quality of rDSD geometries is confirmed by the small values of the Δ*G*EOM contributions, with the possible exception of reactions involving two doublet species (especially HT15 and HT16), where spin contamination effects can become non-negligible also for double-hybrid functionals.^83^ Noted is that geometries optimized by hybrid functionals or MP2 (either UMP2 or ROMP2) methods produce significantly larger Δ*G*EOM contributions.^16,84^

**Table 2:** Geometry (ΔGEOM), Core–Valence (CV), full triples (fT), Perturbative Quadruples (pQ), Diagonal Born–Oppenheimer (DBOC), Relativistic (rel), and Spin–Orbit (ΔSO) Contributions to the Energy Barriers *i*ncluded in the HTBH38/08 Database^a^

	reactions	ΔGEOM	CV^b^	fT	pQ	DBOC	rel	ΔSO
HT1	H^•^ + HCl → H2 + Cl^•^	0.0/1.6	0.1/–0.3 (0.1/–0.3)	–0.3/0.2	–0.1/–0.2	1.6/1.0	–0.5/0.8	0.1/3.5
HT2	OH^•^ + H_2_ → H_2_O + H^•^	0.0/0.0	0.0/0.8 (0.0/0.8)	–0.5/–1.1	–0.3/0.3	0.2/1.1	0.0/–0.6	0.8/0.0
HT3	CH_3_^•^ + H_2_ → CH_4_ + H^•^	0.0/0.0	0.0/0.6 (−0.1/0.6)	–0.2/–0.4	–0.1/–0.1	1.2/1.9	0.0/–0.1	–
HT4	OH^•^ + CH_4_ → CH_3_^•^ + H_2_O	0.0/0.0	0.3/0.4 (0.3/0.4)	–0.1/–0.5	–0.7/–0.1	0.4/0.7	0.0/–0.5	0.8/0.0
HT5	H^•^ + H_2_ → H_2_ + H^•^	–0.1/–0.1	0.0/0.0 (0.0/0.0)	–0.3/–0.3	–0.8/–0.8	1.7/1.7	0.0/0.0	–
HT6	OH^•^ + NH_3_ → H_2_O + NH_2_^•^	–0.2/0.7	0.3/-0.1 (0.4/0.0)	–0.9/–1.0	–1.2/–0.8	1.7/1.5	0.0/–0.2	0.8/0.0
HT7	HCl + CH_3_^•^ → Cl^•^ + CH_4_	0.0/1.7	0.0/0.3 (0.1/0.3)	0.0/0.4	–0.3/–0.3	0.2/0.3	0.0/1.2	0.0/3.5
HT8	OH^•^ + C_2_H_6_ → H_2_O + C_2_H_5_^•^	0.0/0.0	0.2/0.6 (0.2/0.6)	–0.2/–0.4	–0.6/–0.1	–0.3/0.3	–0.2/–0.7	0.8/0.0
HT9	F^•^ + H_2_ → HF + H^•^	0.0/0.0	0.1/0.7 (0.1/0.7)	–0.5/–0.9	–0.2/0.6	0.1/0.9	0.0/–0.8	–
HT10	^3^O + CH_4_ → OH^•^ + CH_3_^•^	–0.4/–1.3	0.4/0.3 (0.4/0.3)	–0.2/–0.1	–0.2/0.2	0.1/–0.1	0.1/–0.3	0.9/0.8
HT11	H^•^ + PH_3_ → PH_2_^•^ + H_2_	0.0/0.0	–0.1/–0.3 (0.0/–0.4)	–0.5/–0.4	–0.1/–0.2	0.9/0.2	–0.2/0.5	–
HT12	H^•^ + OH^•^ →H_2_ + ^3^O	–0.6/0.3	0.4/0.0 (0.4/-0.1)	–0.6/–0.6	0.1/–0.4	1.5/0.9	–0.4/0.1	0.8/0.9
HT13	H^•^ + H_2_S →H_2_ + HS^•^	0.0/0.0	0.0/–0.4 (0.0/–0.5)	–0.4/–0.1	–0.1/–0.3	0.9/0.3	–0.3/0.7	0.0/2.3
HT14	^3^O + HCl → OH^•^ + Cl^•^	–0.7/0.1	0.1/0.2 (0.4/0.4)	–2.4/–1.8	–1.3/–0.9	1.4/1.3	–0.4/0.4	0.9/4.3
HT15	NH_2_^•^ + CH_3_^•^ → CH_4_ + NH	–2.5/–1.2	0.4/0.3 (0.4/0.3)	0.0/0.0	–0.3/–0.6	2.0/2.1	–0.2/0.1	–
HT16	NH_2_^•^ + C_2_H_5_^•^ → NH + C_2_H_6_	–2.6/–1.3	0.5/0.1 (0.5/0.1)	0.3/0.1	–0.4/–0.7	1.1/0.8	–0.2/0.1	–
HT17	NH_2_^•^ + C_2_H_6_ → NH_3_ + C_2_H_5_^•^	0.0/0.0	0.1/0.9 (0.1/0.9)	0.5/0.3	–0.6/–0.5	0.7/1.5	0.0/–0.3	–
HT18	NH_2_^•^ + CH_4_ → NH_3_ + CH_3_^•^	0.0/0.0	0.1/0.7 (0.1/0.7)	0.5/0.2	–0.6/–0.4	0.8/1.2	0.1/–0.3	–
HT19	s-trans cis–C_5_H_8_ → same	0.01/0.1	0.6/0.6 (0.6/0.6)	0.4/0.4	–1.7/–1.7	0.3/0.3	0.0/0.0	–

a All the values are in kJ mol^–1^.

b F12a (F12b).

**Table 3:** Geometry (ΔGEOM), Core–Valence (CV), Full Triples (fT), Perturbative Quadruples (pQ), Diagonal Born–Oppenheimer (DBOC), Relativistic (rel), and Spin–Orbit (ΔSO) Contributions to the Energy Barriers Included in the NHTBH38/08 Database^a^

	reactions	ΔGEOM^b^	CV–F12^c^	fT	pQ	DBOC	rel	ΔSO
NHT1	H^•^ + N_2_O → OH^•^ + N_2_	0.2/–0.9	0.6/0.1 (0.6/0.1)	–0.6/1.9	–0.8/–5.1	1.1/0.4	–0.3/0.4	0.0/0.8
NHT2	H^•^ + FH → HF + H^•^	0.1/0.1	0.5/0.5 (0.5/0.5)	–0.1/–0.1	–0.4/–0.4	2.3/2.8	–0.7/–0.7	–
NHT3	H^•^ + ClH → HCl + H^•^	–0.1/–0.1	0.2/0.2 (0.2/0.2)	–0.4/–0.4	–0.2/–0.2	1.5/1.5	–0.8/–0.8	–
NHT4	H^•^ + FCH_3_ → HF + CH_3_^•^	–0.1/0.0	0.6/1.3 (0.5/1.3)	–0.7/–0.3	–0.7/–0.9	0.7/0.2	–0.7/–0.7	–
NHT5	H^•^ + F_2_ → HF + F^•^	0.8/0.8	0.1/1.6 (0.1/1.6)	–0.2/0.3	0.3/–2.8	0.7/0.3	–0.1/–1.0	–
NHT6	CH_3_^•^ + FCl → CH_3_F + Cl^•^	0.0/0.0	0.3/1.1 (0.3/1.0)	–0.1/0.0	–0.9/–1.7	0.5/0.6	–0.3/–0.7	0.0/3.5
NHT7	F^–^ + CH_3_F → FCH_3_ + F^–^	/	1.5/1.5 (1.5/1.5)	–0.5/–0.5	–0.6/–0.6	0.0/0.0	–0.2/–0.2	–
NHT8	F^–^···CH_3_F → FCH_3_···F^–^	/	1.1/1.1 (1.1/1.1)	–0.3/–0.3	–0.5/–0.5	0.1/0.1	–0.2/–0.2	–
NHT9	Cl^–^ + CH_3_Cl → ClCH_3_ + Cl^–^	/	1.2/1.2 (1.4/1.4)	–0.6/–0.6	–0.5/–0.5	0.0/0.0	–0.2/–0.2	–
NHT10	Cl^–^···CH_3_Cl → ClCH_3_···Cl^–^	/	1.1/1.1 (1.2/1.2)	–0.5/–0.5	–0.5/–0.5	0.0/0.0	–0.5/–0.5	–
NHT11	F^–^ + CH_3_Cl → FCH_3_ + Cl^–^	/	1.4/0.8 (1.5/0.9)	–0.6/–0.5	–0.4/–0.9	0.0/0.0	–0.2/–0.1	–
NHT12	F^–^···CH_3_Cl → FCH_3_···Cl^–^	/	1.0/0.6 (1.0/0.7)	–0.4/–0.4	0.3/–0.4	0.0/0.0	–0.2/–0.3	–
NHT13	OH^–^ + CH_3_F → HOCH_3_ + F^–^	/	1.3/2.0 (1.3/2.0)	–0.3/–0.5	–1.0/–0.9	0.0/0.1	–0.1/–0.5	–
NHT14	OH^–^···CH_3_F → HOCH_3_···F^–^	/	1.1/1.8 (1.1/1.8)	–0.2/–0.6	–0.9/–0.6	0.1/0.1	–0.2/–0.4	–
NHT15	H^•^ + N_2_ → HN_2_^•^	0.0/0.0	0.3/0.4 (0.2/0.4)	–0.5/0.7	1.0/–0.1	1.3/0.4	0.1/–0.4	–
NHT16	H^•^ + CO → HCO^•^	0.0/0.0	0.1/1.0 (0.1/1.0)	–0.4/–0.1	–0.2/–0.1	0.8/–0.1	0.0/–0.4	–
NHT17	H^•^ + C_2_H_4_ → C_2_H_5_^•^	0.0/0.2	0.2/0.0 (0.2/0.0)	–0.5/0.2	0.0/–0.8	0.7/0.0	0.0/–0.2	–
NHT18	CH_3_^•^ + C_2_H_4_ → CH_3_CH_2_CH_2_^•^	–0.1/–0.1	0.6/0.6 (0.6/0.6)	–0.8/–1.0	–0.5/–0.8	0.0/0.0	0.0/–0.2	–
NHT19	HCN → HNC	0.1/0.1	1.6/0.8 (1.7/0.8)	–0.6/0.2	0.4/–0.8	0.1/0.2	–0.3/–0.4	–

a All the values are in kJ mol^–1^.

b the geometries have not been reoptimized at the junChS-F12 level when charged species were involved.

c F12a (F12b).

In more general terms, the results collected in Tables 2 and 3 show that none of the contributions mentioned above can be neglected for reaching fully converged values. As already mentioned, this is also the case for anharmonic corrections to ZPEs (see Δanh in Table 1). In this connection, we recall that accurate ZPEs and thermal contributions can be obtained in the framework of the SPT^68^ from HDCPT2^65^ computations employing rDSD anharmonic force fields.^16,51^ All in all, evaluation of electronic energies at the junChS-F12 level in conjunction with rDSD geometries and vibrational frequencies represents a reliable tool for the study of medium- to large-size systems in the absence of strong multireference effects.

## Rate Constants

In this section, we analyze the performance of three composite schemes (junChS-F12, *Best* and the largely employed CBS-QB3 model^11^) for the computation of rate constants within the ME/AITST approach. To the purpose we have chosen at least one example for each of the main classes of reactions considered in the reference databases discussed in the preceding section. The selected reactions are CH_4_ + OH → CH_3_ + H_2_O (HT4) and H + PH_3_ → PH_2_ + H_2_ (HT11) for hydrogen transfer; Cl^–^ + CH_3_Cl → ClCH_3_ + Cl^–^ (NHT10) for nucleophilic substitution; HCN → HNC (NHT19) for unimolecular isomerization, and H + FH → HF + H (NHT2) for heavy-atom transfer. While the original recipes have been used for the evaluation of ZPE and thermal effects in the CBS-QB3 method, rDSD geometries and VPT2 anharmonic frequencies have been used in junChS-F12 and *Best* computations in order to avoid any empirical scaling factor.

**Figure 2:**
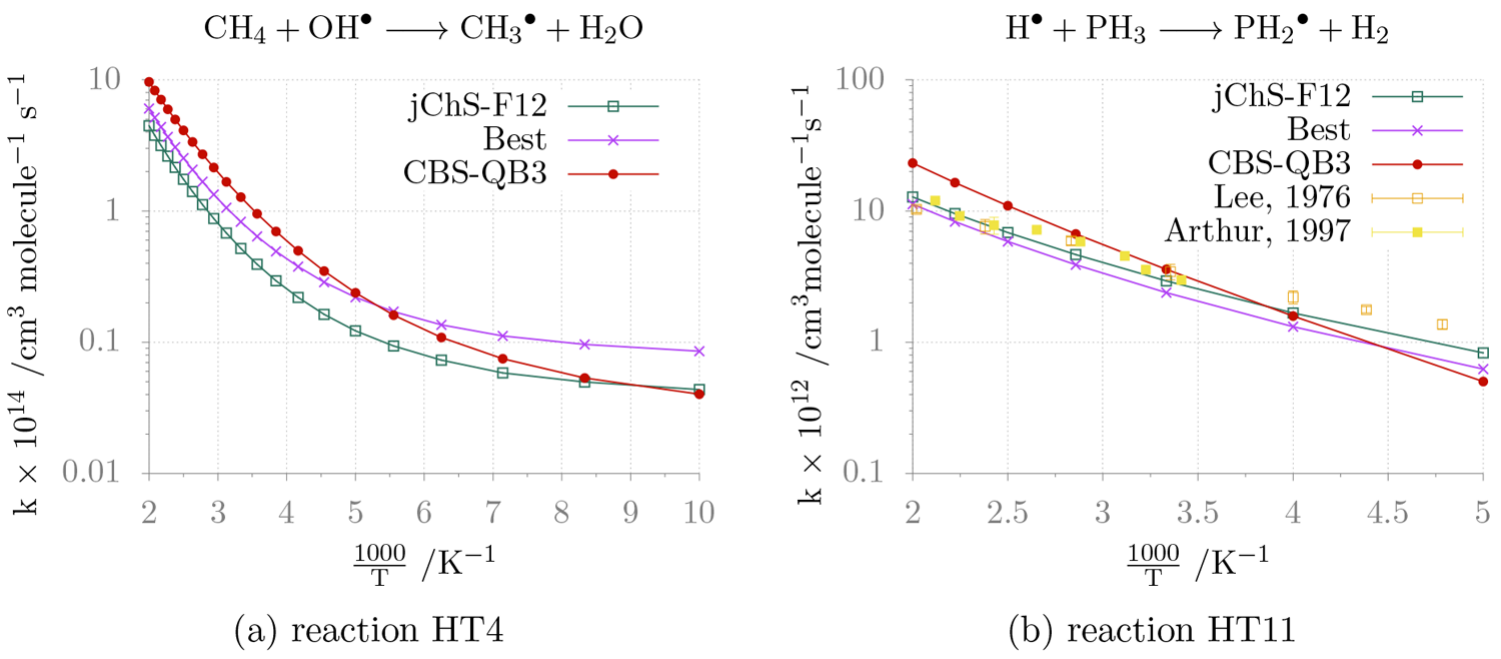
Temperature dependence of the rate constants for reactions HT4 and HT11 in the high-pressure limit.

The reaction of OH with CH_4_ (HT4) is very important in the Earth’s troposphere since it accounts for about 90% of the total CH_4_ sink.^85^ The junChS-F12 and *Best* energy barriers are quite close (27.7 and 28.1 kJ mol^–1^, respectively), whereas a slightly lower value (26.0 kJ mol^–1^ at the W1 level^82^) was estimated in the most exhaustive computation of the rate constant performed until now.^86^ In any case, the reaction shows a strongly non-Arrhenius behavior (related also to the presence of a hindered rotation at the transition state) and the agreement between different model chemistries is only fair at low temperatures (see panel (a) of Figure 2). While a more comprehensive analysis of all the factors (path curvature, anharmonicity, etc.) playing a role in determining the rate constant of this reaction is beyond the scope of the present paper, we point out that the difference between harmonic and anharmonic ZPEs is not negligible (about 1 kJ mol^–1^ for both the forward and backward reaction).

Inspection of panel (b) in Figure 2 shows that reaction between H and PH_3_ (HT11) follows the Arrhenius behavior. The rate constants computed from junChS-F12 and *Best* energy barriers are in remarkable agreement with the available experimental data,^87,88^ whereas the CBS-QB3 energy barrier leads to underestimated rate constants at low temperatures and overestimated rate constants at high temperatures. In this case the role af anharmonicity on the rate constants is negligible: for instance, the difference between harmonic and anharmonic (VPT2) ZPEs is about 0.3 kJ mol^–1^. All these trends are confirmed by the coefficients of the Arrhenius–Kooij fittings collected in Table 4.

**Table 4:** Arrhenius–Kooij Parameters of the Reactions Investigated in the Present Paper

		junChS-F12	*Best*	CBS-QB3
CH_4_ + OH → CH_3_ + H_2_O	*A*/cm^3^ molecule^–1^ s^–1^	3.76 × 10^–14^	3.77 × 10^–14^	8.19 × 10^–14^
	*n*	2.86	2.86	2.56
	*E*_a_/kJ mol^–1^	5.31	4.07	4.74
H + PH_3_ → PH_2_ + H_2_	*A*/cm^3^ molecule^–1^ s^–1^	9.06 × 10^–12^	8.96 × 10^–12^	4.84 × 10^–11^
	*n*	2.02	2.03	1.60
	*E*_a_/kJ mol^–1^	2.85	3.34	6.47
H + HF → HF + H	*A*/cm^3^ molecule^–1^ s^–1^	1.75 × 10^–16^	1.75 × 10^–16^	5.04 × 10^–16^
	*n*	1.81	1.81	1.74
	*E*_a_/kJ mol^–1^	1.57 × 10^2^	1.57 × 10^2^	1.63 × 10^2^
Cl^–^···CH_3_Cl → ClCH_3_···Cl^–^	*A*/s^–1^	1.19 × 10^11^	1.19 × 10^11^	2.34 × 10^11^
	*n*	1.05	1.06	7.56 × 10^–1^
	*E*_a_/kJ mol^–1^	5.10 × 10^1^	5.00 × 10^1^	5.07 × 10^1^
HCN → HNC	*A*/s^–1^	1.13 × 10^14^	8.61 × 10^13^	1.78 × 10^13^
	*n*	6.42 × 10^–2^	1.93 × 10^–1^	1.01
	*E*_a_/kJ mol^–1^	1.88 × 10^2^	1.87 × 10^2^	1.82 × 10^2^
C_2_H_4_ + CN	*A*/cm^3^ molecule^–1^ s^–1^	5.89 × 10^–10^	5.86 × 10^–10^	5.90 × 10^–10^
	*n*	8.55 × 10^–2^	5.05 × 10^–2^	1.05 × 10^–1^
	*E*_a_/kJ mol^–1^	5.45 × 10^–2^	7.73 × 10^–2^	4.18 × 10^–2^
CH_2_OO + H_2_O	*A*/cm^3^ molecule^–1^ s^–1^	2.83 × 10^–15^	–	2.36 × 10^–15^
	*n*	1.05	–	1.12
	*E*_a_/kJ mol^–1^	5.63	–	7.30

**Figure 3:**
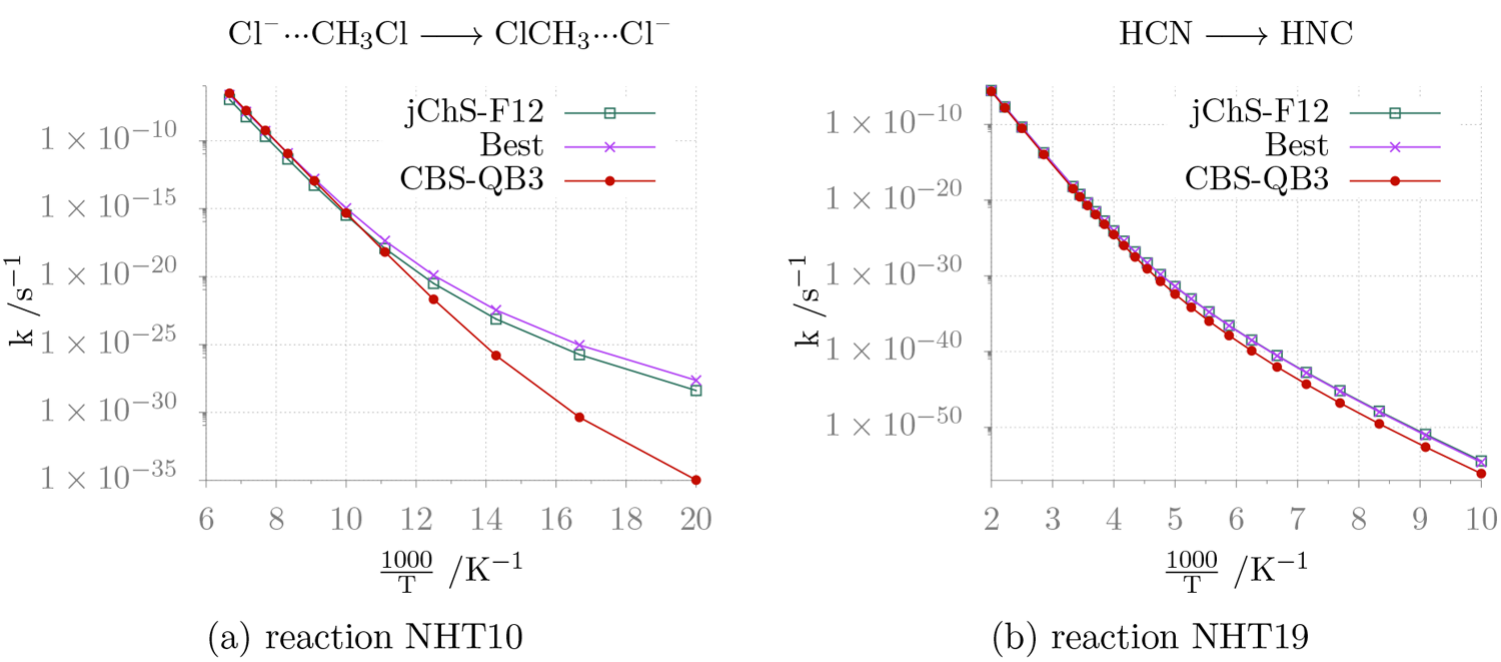
Temperature dependence of the rate constants for reactions NHT10 and NHT19 in the high-pressure limit.

The high pressure limits for the rate constants of reactions NHT10 and NHT19 are shown in Figure 3, while the corresponding Arrhenius-Kooij parameters are given in Table 4. Both reactions are characterized by quite high energy barriers, and their rate constants follow the Arrhenius equation in the medium- to high-temperature range.

Reaction NHT10 is the prototypical S_N_2 reaction, which shows very large environmental effects,^89^ so that accurate computations of gas phase rate constants are the mandatory prerequisite for disentangling intrinsic and environmental effects. The value of the *Best* energy barrier (9.0 kJ mol^–1^) coincides with that obtained from the very accurate focal point approach (FPA) given in ref (90). Since tunneling is expected to play a negligible role, accurate rate constants should be obtained at this level. It is then remarkable that *Best* and junChS-F12 rate constants are very close in the whole range of temperatures, whereas the CBS-QB3 model underestimates significantly the rate constant at low temperatures. In fact, the not too high activation energy leads to a significant deviation from the Arrhenius behavior at low temperatures, with the consequent non-negligible impact of even relatively small errors.

The isomerization of HCN to HNC (NHT19) is of paramount relevance in astrochemistry because the ratio between the two species changes in different environments. While the energy barrier is too high to allow effective isomerization in the interstellar medium (ISM), the rate constant of the reaction represents the reference value for studying catalytic effects by water molecules on icy grains.^91^ In this case, the rate constants computed by different models are in good agreement in the whole temperature range among themselves and with previous computations.^92^

**Figure 4:**
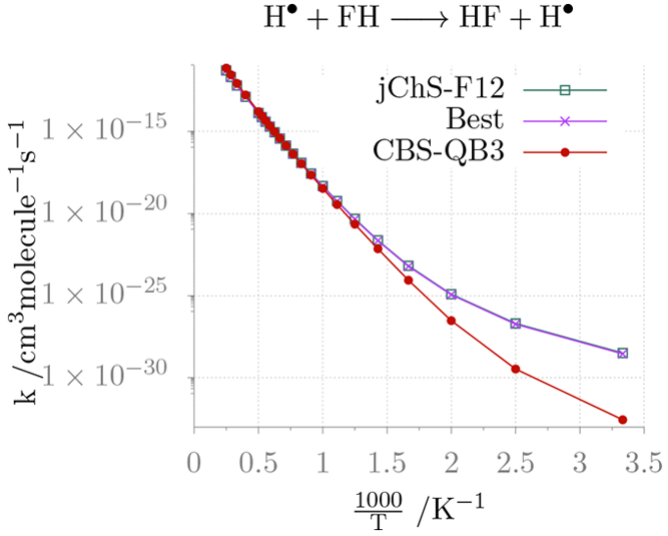
Temperature dependence of the rate constants for reaction NHT2.

The last reaction considered (NHT2) is the simplest heavy atom transfer. Figure 4 shows that also in this case, the rate constants provided by the different methods are in good agreement for medium- to high-temperatures, whereas at low temperatures the CBS-QB3 energy barriers lead to too low rates in comparison with the (close) values of junChS-F12 and *Best* models.

In order to investigate the effect of entrance and exit van der Waals wells, two multistep reactions of astrochemical (CN addition to ethylene) and atmospheric (reaction between the simplest Criegee intermediate and H_2_O) interest have been investigated.

**Figure 5:**
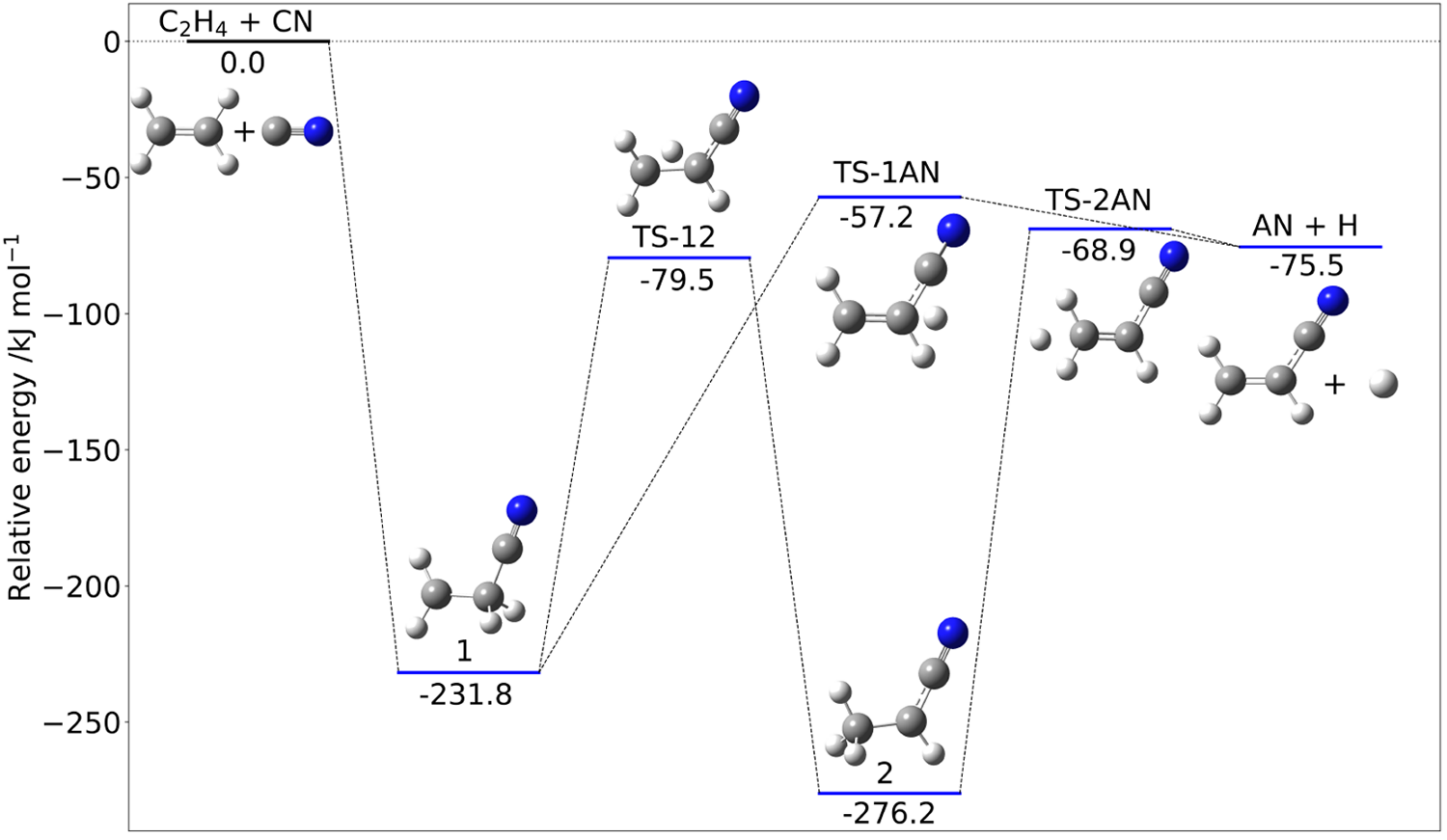
Reaction mechanism for the addition of CN to C_2_H_4_. Electronic energies at the junChS-F12 level augmented by rDSD anharmonic ZPEs.

Aminoacetonitrile (AN), also known as vinylcyanide, has been found in several regions of the ISM^93^ and may be also the best candidate for the formation of cell-like membranes in Titan’s hydrocarbon-rich lakes and seas.^94^ Among the possible formation routes of AN, we have considered the addition of CN radical to ethylene, since both species are present in the ISM and on Titan. From an experimental point of view, the reaction was found to be very fast, approaching the gas kinetics limit at very low and very high temperatures.^95^ On the other hand, the computational studies performed until now did not employ state-of-the-art quantum chemical models.^96^ The reaction mechanism is sketched in Figure 5 together with the junChS-F12 relative energies (including rDSD anharmonic ZPEs) of all the stationary points.

Intermediate 1, which is formed without any entrance barrier, leads to the final products (H and AN), either through a single step ruled by the transition state TS-1AN or by a two-step mechanism involving intermediate 2. In any case, all the energy barriers are submerged, so that this reaction channel is open also in the harsh conditions characterizing the ISM.

**Figure 6:**
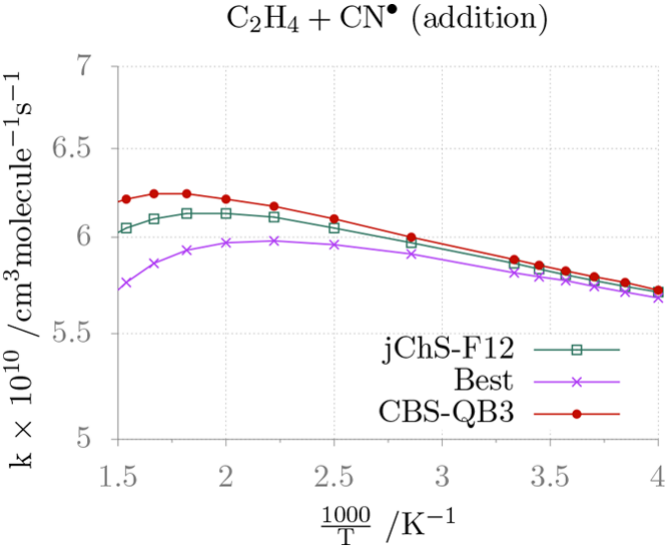
Temperature dependence of the rate constant for the addition of CN to C_2_H_4_ in the high pressure limit.

The rate constant for the addition issued from junChS-F12 computations is compared in Figure 6 with the CBS-QB3 and *Best* counterparts. The addition rate constant is essentially flat in the whole temperature range and the different composite methods provide comparable results. Although a fully quantitative comparison with experimental rate constants is not possible because we have considered only the high pressure limit, our values have the correct order of magnitude, especially at high temperatures.^95^

Criegee intermediates (CIs) are carbonyl oxides formed in the ozonolysis of unsaturated hydrocarbons and play a central role in several processes occurring in the atmosphere. Reaction with water is a key step for several processes involving CIs and has been investigated in a number of studies. In particular, the rate constant for the reaction of the simplest CI (CH_2_OO) has been analyzed in a thorough computational study.^97^

**Figure 7:**
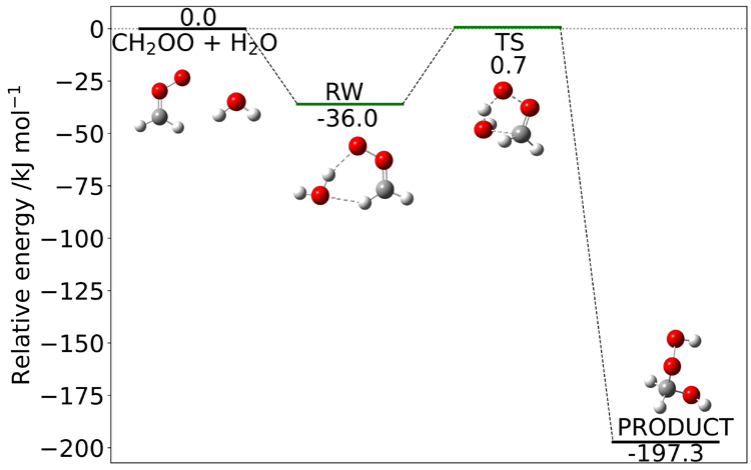
Mechanism of the reaction between CH_2_OO and H_2_O. Electronic energies at the junChS-F12 level augmented by anharmonic rDSD ZPEs.

**Figure 8:**
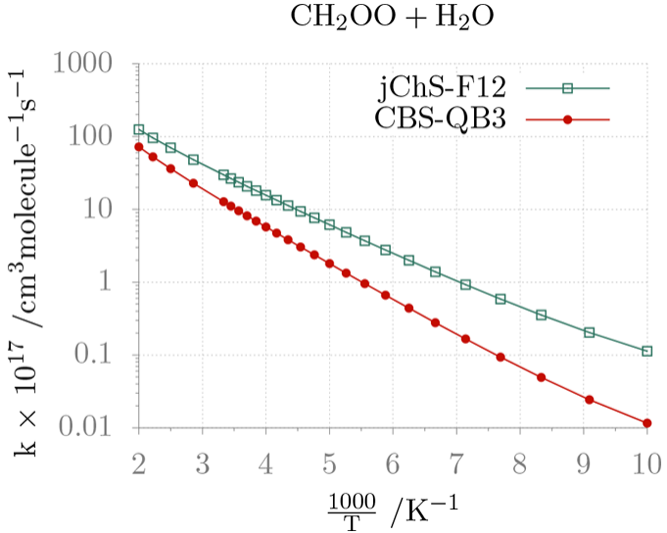
Temperature dependence of the rate constant of the reaction between CH_2_OO and H_2_O in the high pressure limit.

The reaction mechanism is sketched in Figure 7 together with the relative energies of the key stationary points computed at the junChS-F12 level and including anharmonic ZPEs computed at the rDSD level. The rate constants predicted by different composite methods in the framework of the ME/TST model are shown in Figure 8. In this case the temperature dependence of the rate constant is well represented by the simple Arrhenius equation, but the slope is significantly different when employing junChS-F12 or CBS-QB3 energy barriers. Once again all these trends are confirmed quantitatively by the coefficients of the Arrhenius–Kooij fittings given in Table 4 and the junChS-F12 results are in agreement with previous state-of-the-art computations.^97^

## Conclusions

The analysis of processes occurring in nonstandard environments like the atmospheres of exoplanets or the Earth’s troposphere requires accurate kinetic data at low to moderate temperatures and involving barrier heights spanning a large range of values. Furthermore, medium- to large-molecular systems are often involved in those processes, whose entrance channels are tuned by noncovalent interactions. As a consequence, reliable yet effective methods for the computation of rate constants and branching ratios are needed. The master equation formalism employing the ab initio transition state theory offers a reliable reference frame, provided that accurate structural and energetic parameters are available for the key stationary points. To this end, we have validated the recently proposed junChS-F12 model chemistry with reference to very accurate energetic and kinetic data. The results obtained for a large panel of systems and reaction channels show an average error well within the chemical accuracy for all the key thermodynamic and kinetic contributions without the need of any empirical parameter. The junChS-F12 model delivers smaller errors with respect to the reference values than its conventional junChS predecessor, without any excessive increase of computational resources. This behavior can be traced back to the strongly reduced role of CBS extrapolation when going from conventional to explicitly correlated composite methods, with the consequent reduced role of the errors incurred from its estimation by low-order perturbative methods. The computational bottleneck of the proposed model chemistry is the CCSD(T)-F12/jun-cc-pVTZ step. In this connection, new low-scaling approaches^98^ and, possibly, local-correlation models^99,100^ deserve further investigation in order to increase the dimension of molecular systems amenable to accurate computations with reasonable computer requirements. Additional refinements and validations are needed also for situations involving the non-negligible static correlation and/or nonadiabatic effects. However, even taking these limitations into account, we think that the strategy proposed in the present paper can contribute to the computational study of chemical processes under widely different temperature and pressure conditions.

## Supporting Information Available

Additional data for energy barriers (PDF)
