# Supplementary material for: Reliable Gas Phase Reaction Rates at Affordable Cost by Means of the Parameter-Free JunChS-F12 Model Chemistry
Source: J Chem Theory Comput. 2023 May 31;19(12):3526–37. doi: 10.1021/acs.jctc.3c00343 (PMC10308586; doi:10.1021/acs.jctc.3c00343)
Supplement: Supplementary file 1 — ct3c00343_si_001.pdf [file ct3c00343_si_001.pdf]

Supporting Information: Reliable Gas Phase Reaction Rates at Affordable Computer Cost by Means of the Parameter-Free JunChs-F12 Model Chemistry

Vincenzo Barone,* Jacopo Lupi, Zoi Salta, and Nicola Tasinato

Scuola Normale Superiore di Pisa, piazza dei Cavalieri 7, 56125 Pisa, Italy

E-mail: vincenzo.barone@sns.it

Table S1: Energy barriers for the forward and reverse reactions in the HTBH38/08 and NHTBH38/08 datasets (excluding those contained in the DBH24/08 one) obtained by different computational models. All the values (zero-point exclusive) are in $kJ\,mol^{-1}$.

Label	Reaction	forward/reverse barrier height			
		CC-F12	CC-F12+CV	junChS-F12	*Best*
HT1	$H^\bullet + HCl \longrightarrow H_2 + Cl^\bullet$	22.3/32.3	22.4/32.0	21.6/32.1	22.6/30.7
HT2	$OH^\bullet + H_2 \longrightarrow H_2O + H^\bullet$	23.5/90.4	23.4/91.0	23.6/90.7	21.5/91.1
HT3	$CH_3{}^\bullet + H_2 \longrightarrow CH_4 + H^\bullet$	50.1/62.0	50.2/62.5	50.5/62.0	50.0/63.6
HT5	$H^\bullet + H_2 \longrightarrow H_2 + H^\bullet$	40.7/40.7	40.7/40.7	40.5/40.5	40.7/40.7
HT6	$OH^\bullet + NH_3 \longrightarrow H_2O + NH_2{}^\bullet$	14.2/57.5	14.2/57.2	16.9/60.2	16.7/59.5
HT7	$HCl + CH_3{}^\bullet \longrightarrow Cl^\bullet + CH_4$	7.7/29.5	7.6/29.6	7.5/29.5	8.4/30.1
HT8	$OH^\bullet + C_2H_6 \longrightarrow H_2O + C_2H_5{}^\bullet$	16.2/85.7	16.3/86.3	16.5/86.9	14.2/85.5
HT9	$F^\bullet + H_2 \longrightarrow HF + H^\bullet$	7.5/141.0	7.5/141.5	7.6/141.0	5.3/141.5
HT10	$^3O + CH_4 \longrightarrow OH^\bullet + CH_3{}^\bullet$	60.5/37.4	61.0/37.8	61.2/38.7	59.4/37.2
HT11	$H^\bullet + PH_3 \longrightarrow PH_2{}^\bullet + H_2$	12.9/105.1	12.8/105.5	12.3/105.9	12.1/102.6
HT14	$^3O + HCl \longrightarrow OH^\bullet + Cl^\bullet$	44.5/43.2	44.3/43.0	43.7/43.1	42.0/41.6
HT15	$NH_2{}^\bullet + CH_3{}^\bullet \longrightarrow CH_4 + NH$	36.6/92.1	37.1/92.5	37.6/92.3	38.7/93.1
HT16	$NH_2{}^\bullet + C_2H_5 \longrightarrow NH + C_2H_6$	38.5/79.5	39.2/79.7	39.6/79.4	42.0/81.2
HT17	$NH_2{}^\bullet + C_2H_6 \longrightarrow NH_3 + C_2H_5{}^\bullet$	47.6/73.7	47.7/74.7	47.8/75.0	48.5/76.9
HT18	$NH_2{}^\bullet + CH_4 \longrightarrow NH_3 + CH_3{}^\bullet$	58.4/70.0	58.6/70.8	58.7/71.1	60.7/73.9
HT19	s-trans cis$-C_5H_8 \longrightarrow$ same	166.2/166.2	166.4/166.4	166.3/166.3	166.5/166.5
NHT2	$H^\bullet + FH \longrightarrow HF + H^\bullet$	175.2/175.2	175.7/175.7	175.2/175.2	175.6/175.6
NHT4	$H^\bullet + FCH_3 \longrightarrow HF + CH_3{}^\bullet$	126.5/237.2	127.2/239.0	127.8/240.2	124.5/236.1
NHT5	$H^\bullet + F_2 \longrightarrow HF + F^\bullet$	19.2/452.1	19.2/453.8	18.9/452.6	10.8/442.5
NHT7	$F^- + CH_3F \longrightarrow FCH_3 + F^-$	-2.5/-2.5	-1.1/-1.1	-0.9/-0.9	-2.5/-2.5
NHT8	$F^-\cdots CH_3F \longrightarrow FCH_3\cdots F^-$	55.4/55.4	56.5/56.5	56.6/56.6	55.8/55.8
NHT9	$Cl^- + CH_3Cl \longrightarrow ClCH_3 + Cl^-$	11.5/11.5	12.4/12.4	11.0/11.0	10.2/10.2
NHT11	$F^- + CH_3Cl \longrightarrow FCH_3 + Cl^-$	-50.9/82.5	-49.5/82.8	-50.5/83.5	-52.1/83.2
NHT14	$OH^-\cdots CH_3F \longrightarrow HOCH_3\cdots F^-$	45.9/199.0	46.9/200.9	47.6/199.3	47.1/198.9
NHT16	$H^\bullet + CO \longrightarrow HCO^\bullet$	14.1/95.4	14.1/14.1	13.7/95.7	13.6/95.6
NHT18	$CH_3{}^\bullet + C_2H_4 \longrightarrow CH_3CH_2CH_2{}^\bullet$	26.8/138.1	26.9/138.5	27.1/137.4	25.6/136.2
